# Copolymacrolactones Grafted with l-Glutamic Acid: Synthesis, Structure, and Nanocarrier Properties

**DOI:** 10.3390/polym12040995

**Published:** 2020-04-24

**Authors:** Ernesto Tinajero-Díaz, Antxon Martínez de Ilarduya, Sebastián Muñoz-Guerra

**Affiliations:** Departament d´Enginyeria Química, Universitat Politècnica de Catalunya, ETSEIB, Diagonal 647, 08028 Barcelona, Spain; ernesto.tinajero@gmail.com (E.T.-D.); antxon.martinez.de.ilarduia@upc.edu (A.M.d.I.)

**Keywords:** macrolactones ROP, pentadecanolactone ROP, globalide ROP, macrolactones copolyesters, polyester-polypeptide graft copolymers, polyglutamic grafted polyesters, macrolactones copolyesters, unsaturated copolymacrolactones, polyglutamic grafted copolymacrolactones, macrolactones nanoparticles

## Abstract

The enzymatic ring-opening copolymerization (eROP) of globalide (Gl) and pentadecalactone (PDL) was performed in solution from mixtures of the two macrolactones at ratios covering the whole range of comonomeric compositions. The resulting P(Gl_x_-*r*-PDL_y_) random copolyesters were aminofunctionalized by thiol-ene reaction with aminoethanethiol. ROP of γ-benzyl-l-glutamate *N*-carboxyanhydride initiated by P(Gl_x_-*r*-PDL_y_)-NH_2_ provided neutral poly(γ-benzyl-l-glutamate)-grafted copolyesters, which were converted by hydrolysis into negatively charged hybrid copolymers. Both water-soluble and nonsoluble copolymers were produced depending on copolymer charge and their grafting degree, and their capacity for self-assembling in nano-objects were comparatively examined. The emulsion solvent-evaporation technique applied to the chloroform-soluble copolymers grafted with benzyl glutamate rendered well-delineated spherical nanoparticles with an average diameter of 200–300 nm. Conversely, micellar solutions in water were produced from copolyesters bearing grafted chains composed of at least 10 units of glutamic acid in the free form. The copolymer micelles were shown to be able to load doxorubicin (DOX) efficiently through electrostatic interactions and also to release the drug at a rate that was markedly pH dependent.

## 1. Introduction

Polypeptides coming from either natural or synthetic sources are regarded as a class of highly refined polymers closely related with nature. Combination of synthetic polymers with polypeptides commonly results in hybrid copolymers in which the properties of each component are profited to surmount their individual limitations [1]. Thus, the peptide component will provide functionality to the conjugate, whereas the synthetic polymer may increase the stability of the system as well as improve its solubility and biocompatibility. The synthetic polymer is also able of introducing the amphiphilic character required for self-assembly in aqueous environments, and it can even modulate the polypeptide activity [2]. Polyesters are well recognized as polymeric counterparts that can be effectively attached to polypeptides to afford a large family of hybrid materials with outstanding properties as biomaterials [3]. The excellent complementarity of these two types of polymers together with their good accessibility through ring-opening polymerization [4,5] explains why the polyester–polypeptide copolymers are today one of the preferred hybrid systems under study.

Macrolactones (MLs) stand out as ideal monomers for green polymer chemistry [6]. MLs may be polymerized by different ring-opening methods including those that are catalyzed by enzymes. A number of macrolactones are naturally occurring or come from renewable materials. Oxacyclohexadecan-2-one (ω-pentadecalactone, PDL) and oxacyclohexadecen-2-one (globalide, Gl) are 16-membered macrolactones that only differ to each other in that the latter contains a double bond on the 12 cyclic chain carbon. These two MLs are commercially available because of their traditional use in perfumery [7]. The possibilities of PDL as monomer has been widely explored along the last two decades for the synthesis of aliphatic polyesters and copolyesters with high potential as biodegradable and bioresorbable medical materials, whereas such interest for Gl has been moderate. Copolymerization by ROP of PDL with other lactones constitutes the major approach applied for the design of highly hydrophobic polyesters with adjusted thermal and mechanical properties [8,9,10,11,12,13,14,15,16]. A substantial limitation of the polyesters made of PDL is, however, their lack of chemical functionality other than that associated to chain ends. Dove et al. [17] functionalized PPDL by copolymerization with an unsaturated derivative of ε-caprolactone (dihydrocarvide). The resulting block copolyester was capable of undergoing postpolymerization modification but the synthesis was long and tedious. 

The double bond present in Gl provides a versatile tool for chemical functionalization. Gl has been polymerized on several occasions to render polyglobalide (PGl), an unsaturated polyester [18] that was then used to produce cross-linked polymers [19,20] or polypeptide-grafted copolymers [21]. The work here reported deals with the copolymerization of the two MLs, Gl and PDL, with the purpose of producing unsaturated copolyesters suitable for grafting with polypeptides. The resulting polypeptide–polyester graft copolymers will display remarkable amphiphilic character and will be of interest for their potential in the design of drug nanocarriers. The copolymerization PDL with a similar Gl unsaturated ML (ω-6-hexadecenlactone) has been collaterally explored in a recent work devoted to adjusting the thermal behavior of polyesters with a structure close to polyethylene by copolymerization of lactones of different size [22]. Several reasons support the choice of this approach: (a) highly hydrophobic copolyesters with tuned crystallinity and functionality will be feasible by adjusting the content of saturated and unsaturated macrolactones, (b) application of the enzymatic ring-opening polymerization (eROP) to the bio-based ester and peptide building blocks will allow a basically green synthetic procedure suitable for the design of many hybrid copolymers, (c) the bio-based hybrid system that is generated will exhibit a high amphiphilic character as a consequence of the strong contrast between the great hydrophobicity of the long polyester counterpart and the polar nature of the polypeptide side chain, in particular, when the amino acid is in the charged state, and (d) the nanoparticles built from these copolymers should be expected to be highly stable not only due to the occurrence of strong hydrophobic interactions but also due to the tightening effect exerted by the crystallized polyester segments. The particles bearing charged amino acid units will show an efficient loading and pH-dependent release of charged drugs.

## 2. Experimental Section

### 2.1. Materials

Triphosgene and α-pinene were supplied by Sigma-Aldrich and γ-benzyl-l-glutamate (BLG) from Bachem, and 2,2′-Azobis(2-methylpropionitrile) (AIBN) (98%) and HBr (33% *w*/*w* in acetic acid) were acquired from Sigma-Aldrich (St. Louis, MI, United States). Globalide (Gl) was provided by Grupo Indukern (Barcelona), and ω-pentadecalactone (PDL) was purchased from Sigma-Aldrich and distilled under vacuum previous to use. Novozyme 435 (*Candida antarctica* Lipase B immobilized on cross-linked polyacrylate beads) was a gift of Novozymes (Bagsværd, Denmark), and 2-(Boc-amino)ethanethiol (BAET) (97%) and Sodium trifluoroacetate (98%) were purchased from Sigma-Aldrich and used without further purification. Anhydrous DMF (99.8%), CHCl_3_ (99.9%), THF (99.9%), ethyl acetate (99.9%), and *n*-heptane were purchased from Across Organics (Geel, Belgium) and used directly from the bottle under an inert atmosphere. Additionally, 1,1,1,3,3,3-hexafluoro-2-propanol (HFIP) was purchased from Apollo Scientific (UK), and doxorubicin hydrochloride (DOX·HCl) (98%) was purchased from AK Scientific Inc. (Union City, CA, USA).

### 2.2. Characterization

^1^H and ^13^C NMR spectra were recorded on a Bruker AMX-300 spectrometer (Billerica, MA, USA) at 25 °C, operating at 300.1 and 75.5 MHz, respectively. Compounds were dissolved in deuterated chloroform (CDCl_3_) or a mixture of trifluoroacetic acid (TFA) and CDCl_3_, and spectra were internally referenced to tetramethylsilane (TMS). About 10 mg of sample in 1 mL of solvent were used, and 64 scans were recorded for ^1^H NMR. FTIR measurements were performed in a Frontier Perkin-Elmer FTIR spectrophotometer (Waltham, MA, USA). Spectra were obtained from powder samples using a Universal ATR sampling accessory. For spectra recorded at variable temperature, films were prepared by casting an HFIP solution containing 5 mg of polymer in 1 mL of solvent on a NaCl plate and the plate set on a VT FTIR Cell (Specac). Eight scans with a resolution of 4 cm^−1^ were collected for each sample in the spectral region of 450–4000 cm^−1^.

Molecular weight analysis was performed by gel permeation chromatography (GPC) on a Waters equipment provided with RI and UV detectors. Precisely, 100 µL of 0.1% (*w*/*v*) sample solution was injected and chromatographed with a flow of 0.4 mL·min^−1^ using as solvent HFIP with sodium trifluoroacetate added (0.05 M). HR5E and HR2 Waters linear Styragel columns (7.8 mm × 300 mm, pore size 10^3^–10^4^ Å) packed with cross-linked polystyrene and protected with a precolumn were used. Molar mass averages and distributions were calculated against PMMA standards.

Thermogravimetric analyses were performed on a Mettler-Toledo TGA/DSC 1 Star System under a nitrogen flow of 20 mL·min^−1^ at a heating rate of 10 °C·min^−1^ within a temperature range of 30–600 °C. The thermal behavior of cyclic compounds and polymers was examined by differential scanning calorimetry (DSC) using a Perkin-Elmer DSC 8500 apparatus. Thermograms were recorded from 4 to 6 mg samples at heating and cooling rates of 10 °C·min^−1^ under a nitrogen flow of 20 mL·min^−1^. Indium and zinc were used as standards for temperature and enthalpy calibration.

Dynamic light scattering studies (DLS) were performed at the Parc Científic de Barcelona using a Zetasizer Nano ZS series Malvern instrument equipped with a 4 mW He–Ne laser operating with a wavelength of 633 nm. The noninvasive backscatter optic arrangement was used to collect the light scattered by the particles at an angle of 173°. The samples were analyzed in disposable cuvettes at 25 °C.

Real-time X-ray diffraction (XRD) studies were carried out using synchrotron radiation at the BL11 beamline for noncrystalline diffraction (NCD) at ALBA (Cerdanyola del Vallès, Barcelona, Spain). Spectra were recorded from powder samples subjected to heating–cooling cycles at a rate of 10 °C·min^−1^. The energy employed corresponded to a 0.10 nm wavelength, and the spectra were calibrated with silver behenate (AgBh) and Cr_2_O_3_.

### 2.3. Synthesis

**γ-Benzyl L-glutamate N-carboxyanhydride (BLG-NCA):** γ-benzyl L-glutamate (10 g, 42.14 mmol), α-pinene (11.4 g, 84.2 mmol), and ethyl acetate (80 mL) were weighed in a three-neck round-bottom flask and refluxed under nitrogen for 15 min. Then, triphosgene (6.25 g, 21.05 mmol) dissolved in ethyl acetate (20 mL) was added dropwise and refluxing continued until most of the solids disappeared which took about 3 h. The reaction mixture was then cooled down and filtered, and the filtrate was reduced to approximately 30% of its original volume by rotaevaporation. BLG-NCA was precipitated by addition of heptane (60 mL), recovered by filtration under vacuum, recrystallized twice using an ethyl acetate/heptane mixture (90:10 *v*/*v*), washed with heptane, and finally dried under vacuum. Yield: 90%.

**Synthesis of P(Gl_x_-*r*-PDL_y_) copolyesters by eROP:** Precisely, 400 mg of Novozyme 435 (CALB, 20% *w*/*w*) was placed in a round-bottom flask and dried over P_2_O_5_ for 16 h at 50 °C in a desiccator. Dry toluene (7 mL) and 2 g of the mixture of the two MLs at the selected molar ratio (Gl/PDL: 0/100, 10/90, 30/70, 50/50, 70/30, 90/10, and 100/0) were then added. The flask was immediately immersed in an oil bath at 70 °C, and the reaction left to proceed for 24 h under stirring in a nitrogen atmosphere. Finally, the reaction mixture was cooled down and toluene was removed by rotaevaporation. The solid residue was dispersed in chloroform, and the enzyme was filtered out. The polymer was precipitated by pouring the filtered chloroform solution into cold methanol, recovered by filtration, and dried before characterization. Yield: 80%–90%. 

**Functionalization of the P(Gl_x_-*r*-PDL_y_) copolyesters via thiol-ene reaction:** P(Gl_13_-*r*-PDL_87_) and P(Gl_48_-*r*-PDL_52_) copolyesters were chosen for the synthesis of the grafted copolymers. Briefly, the corresponding copolyester (0.2 g), BAET (1.11 g), and AIBN (50 mg) were weighed in a Schlenk tube purged with nitrogen, and then, 1 mL of THF was added. The reaction was initiated by immersing the tube into an oil bath at 80 °C and left to proceed for 24 h under magnetic stirring. The reaction was terminated by addition of dichloromethane (DCM) and immersion of the tube in an ice bath. The reaction product was precipitated in cold methanol and recovered by centrifugation. This process was performed twice to render the BAET-modified copolymers P[(Gl-BAET)_13_-*r*-PDL_87_] and P[(Gl_24_-*r*-(Gl-BAET)_24_-*r*-PDL_52_]. Yield: 90%.

**Removal of Boc-protecting groups:** Solutions of copolyesters, P[(Gl-BAET)_13_-*r*-PDL_87_] and P[Gl_24_-*r*-(Gl-BAET)_24_-*r*-PDL_52_], (100 mg in 2 mL of TFA), were stirred at room temperature for 10 min. The solution was then added to an excess of diethyl ether, and the precipitate was then recovered by centrifugation and washed twice with a saturated 0.5 M NaHCO_3_ aqueous solution. The products, P[(Gl-NH_2_)_13_-*r*-PDL_87_] and P[Gl_24_-*r*-(Gl-NH_2_)_24_-*r*-PDL_52_], were dried under vacuum at room temperature and stored under such conditions until being used. Yield: 90%.

**Synthesis of P[(Gl_x_-*r*-PDL_y_)–*g*–BLG_z_] graft copolymers:** Graft copolymers were prepared by ROP of BLG-NCA using P[(Gl-NH_2_)_13_-*r*-PDL_87_] and P[Gl_24_-*r*-(Gl-NH_2_)_24_-*r*-PDL_52_] as multiple macroinitiators. To prepare P[(Gl_13_-*r*-PDL_87_)–*g*–BLG_10_], the P[(Gl-NH_2_)_13_-*r*-PDL_87_] copolymer (5.2 mg, 0.016 mmol) in 2 mL of dried CHCl_3_ was injected with a syringe into a Schlenk tube through a rubber septum containing a solution of BLG-NCA (290 mg, 1.10 mmol) in 6 mL of dry CHCl_3_. The tube was placed in a NaCl water bath at 0 °C, and the reaction was left to proceed under stirring for 48 h or until the BLG-NCA was completely consumed as monitored by FTIR spectroscopy. The final reaction mixture was poured into an excess of diethyl ether, and the precipitate was recovered by centrifugation and dried under vacuum. The same methodology was used to prepare P[(Gl_48_-*r*-PDL_52_)–*g*–BLG_2_] copolymer from P[Gl_24_-*r*-(Gl-NH_2_)_24_-*r*-PDL_52_] (6.02 mg, 0.019 mmol) and BLG-NCA (100 mg, 0.382 mmol) but using DMF as solvent instead of CHCl_3_. Yield: 80%–90%.

**Graft copolymer deprotection:** A general procedure was used for deprotection of PBLG pendant groups. Briefly, a solution of 170 mg of P[(Gl_13_-*r*-PDL_87_)–*g*–BLG_10_] in 2 mL of TFA was first prepared. Then, a solution of HBr in glacial acetic acid in a molar excess of 2.5–5 times respect to the γ-benzyl L-glutamate repeating unit was added slowly to the copolymer solution at 0 °C, and after 2 h, the mixture was poured into an excess of diethyl ether. The precipitate was centrifuged and washed twice with diethyl ether. The obtained polymer was dissolved in 0.5 M NaHCO_3_ aqueous solution and then dialyzed (MWCO 2000) against distilled water to yield P[(Gl_13_-*r*-PDL_87_)–*g*–LGA_10_] in the salt form. P[(Gl_48_-*r*-PDL_52_)–*g*–BLG_2_] was treated in the same way to yield P[(Gl_48_-*r*-PDL_52_)–*g*–LGA_2_] in 70% yield. 

**Synthesis of poly(γ-benzyl-L-glutamate) (PBLG) and poly(l-glutamic acid) (PLGA):** γ-benzyl-l-glutamate NCA (800 mg, 3.03 mmol) was dissolved in 7 mL of anhydrous DMF in a Schlenk tube. The tube was immersed in a 0 °C NaCl water bath, and 80 µL (6.1 mg, 0.0606 mmol) of a stock solution 0.76 M of hexylamine (HA) in DMF was injected through a rubber septum with a syringe ([NCA]_0_/[HA]_0_ = 50). The reaction was left until the BLG-NCA had been completely consumed as monitored by FTIR spectroscopy. After complete monomer conversion, the polypeptide was precipitated into an excess of chilled diethyl ether, filtered, and dried under vacuum. Yield: 84%. Removal of benzyl groups from PBLG was carried out in a similar manner as in the graft copolymer P[(Gl_13_-*r*-PDL_87_)–*g*–(BLG)_10_] to produce PLGA in 70% yield. 

### 2.4. Graft Copolymer Self-Assembling

Spherical nanoparticles (NPs) made of P[(Gl_13_-*r*-PDL_87_)–*g*–BLG_10_] copolymer were prepared by the emulsion-solvent evaporation technique. Precisely, 10 mg of the copolymer was dissolved in 2 mL of chloroform, and the solution was added to 10 mL of 1% (*w*/*w*) poly(vinyl alcohol) (PVA) (*M*_n_ = 2000 g·mol^−1^) aqueous solution. The mixture was then sonicated thrice for 15 s each time to yield a homogeneous oil-in-water emulsion. This emulsion was immediately poured into 10 mL of 0.3% PVA aqueous solution and magnetically stirred in an open beaker at room temperature for 3 h to evaporate the organic solvent. The NPs were collected by centrifugation at 11,000 rpm and washed three times with distilled water prior to characterization.

### 2.5. Doxorubicin Loading and Releasing

Doxorubicin hydrochloride (DOX·HCl) was used as a drug model to appraise the capacity of P[(Gl_13_-*r*-PDL_87_)–*g*–LGA_10_] to load positively charged drugs by electrostatic conjugation. Briefly, 5 mg of the copolymer was solubilized in 4 mL of deionized water, and the solution (pH 7.0) was stirred for 10 min before passing it through a 0.45 μm filter. Then, DOX·HCl (3, 1, or 0.5 mg) dissolved in water (1 mL) was added dropwise into the polymer–aqueous mixture under magnetic stirring (300 rpm) and left under stirring for 12 h. Afterward, the mixture was dialyzed (Spectra/Por membrane tubing; MWCO 2000 kDa, Spectrum Labs) for 24 h against distilled water to remove the free DOX·HCl, and the dialysate was then lyophilized. Drug loading efficiency (DLE) and drug loading content (DLC) were calculated according to the following equations:

$$\mathrm{DLE}\%=\frac{\mathrm{Mass}\;\mathrm{of}\;\mathrm{the}\;\mathrm{drug}\;\mathrm{in}\;\mathrm{NPs}}{\mathrm{Mass}\;\mathrm{of}\;\mathrm{the}\;\mathrm{drug}\;\mathrm{in}\;\mathrm{feed}}\times 100\;\;\;\;\;\;\;\;\;\;\;\mathrm{DLC}\%=\frac{\mathrm{Mass}\;\mathrm{of}\;\mathrm{drug}\;\mathrm{in}\;\mathrm{NPs}}{\mathrm{Mass}\;\mathrm{of}\;\mathrm{NPs}}\times 100$$

The effect of the pH on drug release was assessed by incubating the DOX-loaded NPs in following buffers: PBS pH 7.4, citrate–phosphate pH 4.2, and hydrochloric acid–potassium chloride pH 2.0. The DOX·P[(Gl_48_-*r*-PDL_52_)–*g*–LGA_10_] conjugate was placed in a dialysis bag (Spectra/Por membrane tubing; MWCO 6000–8000 kDa, Spectrum Labs) which was then immersed in 25 mL of buffer and kept under constant mild shaking at 37 °C. For measuring the amount of drug released, 1.5 mL aliquots were taken out from the releasing medium at selected time intervals, and the solution was replenished with an equal volume of fresh medium. Quantification of DOX was accomplished by absorption spectrometry at 480 nm using a UV–Vis spectrophotometer.

## 3. Results and Discussion

### 3.1. PGl-r-PDL Copolyesters

**Scheme 1:**
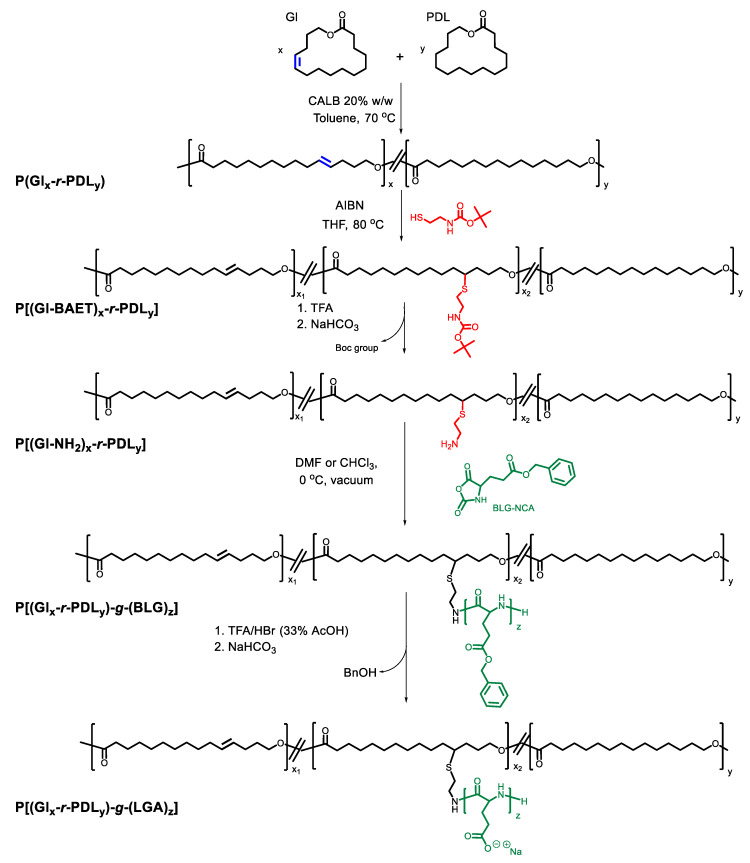
Synthesis of P[(Gl_x_-*r*-PDL_y_)–*g*–(LGlu)_z_] copolymers.

The set of reactions leading to the synthesis of the l-Glu-grafted poly(globalide-*random*-pentadecalactone) copolyesters with the l-Glu either protected as benzyl ester (BLG) or with the carboxyl groups in the free form, namely, P[(Gl_x_-*r*-PDL_y_)–*g*–(BLG)_z_] and P[(Gl_x_-*r*-PDL_y_)–*g*–(LGA)_z_], respectively, are depicted in Scheme 1. First, a series of P(Gl_x_-*r*-PDL_y_) random copolyesters made of globalide (Gl) and pentadecalactone (PDL) was prepared by enzymatic ring-opening polymerization (eROP) using *Candida antarctica* Lipase B (CALB, Novozyme 435). The results obtained in these copolymerizations are shown in Table 1. The eROP method, previously used for the homopolymerization of both Gl and PDL [23,24,25], has afforded satisfactory results in the copolymerization of these two MLs with yields between 75% and 90% and copolymer compositions showing small deviations with respect to feed compositions. The copolymerization of 6-hexadecenlactone and PDL described recently by Pappalardo et al. [22] carried out in the presence of a pyridylamidozinc(II) complex rendered copolymers with essentially the same compositions as that of their feeds. NMR was used for the chemical characterization of the copolyesters including composition and chain length. The ^1^H and ^13^C NMR spectra of P(Gl_13_-*r*-PDL_87_) are given in Figure S1 (in Supplementary Materials), and ^1^H NMR spectra for the whole series are compared in Figure S2 (in Supplementary Materials). Since GPC analysis of P(Gl_x_-*r*-PDL_y_) was difficult due to their scarce solubility in the solvents commonly used as eluents, their chain sizes were estimated by end-group NMR analysis. The area of signals due to CH_2_OH end groups appearing at 3.6 ppm (peak “d” in Figure S1) was compared with the area of CH_2_OOC appearing at 4.2 ppm (peaks d and d’), and assuming that chains were ended by both hydroxyl and carboxyl end groups, the degree of polymerization was estimated. Number-average molecular weights (*M*_n_) oscillating in the 9000–12,000 g·mol^−1^ range without showing apparent correlation with composition were measured. Determination of comonomers distribution along the copolymer chain was unfeasible because NMR spectra were scarcely sensitive to sequence distribution effects given the long distance between ester groups and also extremely complex due to the existence of both constitutional and geometric isomerism. In fact, the Gl sample used in this work consisted of a 60/40 mixture of two monounsaturated isomers corresponding to the double bond placed at either 11- or 12-position with an overall diastereomeric E/Z configuration ratio of 78/22. ^13^C NMR spectra of Gl and PGl are compared in Figure S3 (in Supplementary Materials) indicating that the isomeric ratio present in the macrolactone was retained in the copolyesters generated by eROP. Nevertheless, a random distribution of the two comonomers is assumed to be present in these copolymers according to what should be expected from the well-known indiscriminate transesterase activity of lipases [26], and also in agreement with the microstructure generated in the eROP of a diversity of PDL-based copolyesters that have been previously reported [10,11,13,14].

The thermal stability of P(Gl_x_-*r*-PDL_y_) copolyesters in the absence of oxygen was evaluated by TGA along the 30–600 °C interval under a circulating nitrogen flow. Their TGA traces along with those recorded for the two homopolyesters, PGl and PPDL, as well as their respective derivative curves are shown in Figure S4 (Supplementary Materials). Relevant parameters, the onset and maximum rate decomposition temperatures and remaining weights, as they were estimated in these assays are listed in Table 2. The thermal decomposition pattern displayed by the homopolyesters and copolyesters is similar, as it is indicated by resemblance between their respective derivative curves. Inspection of the TGA data indicates that decomposition of all copolymers appeared to take place through two main steps confined in the 400–500 °C range with *^max^T_d_* values closely around those of PGl and PPDL. As it is characteristic of most of aliphatic polyesters and according to what should be expected from the high resistance to heat displayed by PGl and PPDL, the TGA data collected in this study ascertain the great thermal stability of the P(Gl_x_-*r*-PDL_y_) copolyesters and reveal their ability to decompose cleanly without hardly leaving residual product.

**Figure 1:**
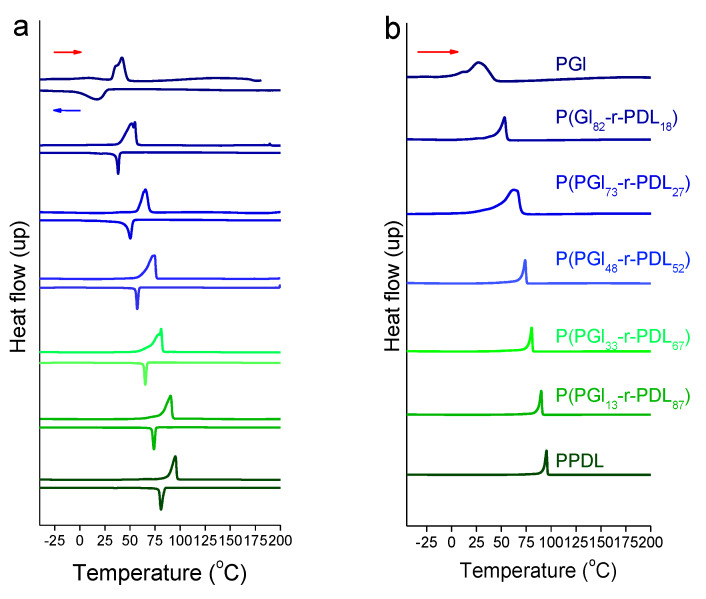
Differential scanning calorimetry (DSC) traces of P(Gl_x_-*r*-PDL_y_) copolyesters recorded at the first heating and subsequent cooling (**a**) and the second heating (**b**) in the −30 to 200 °C range.

**Figure 2:**
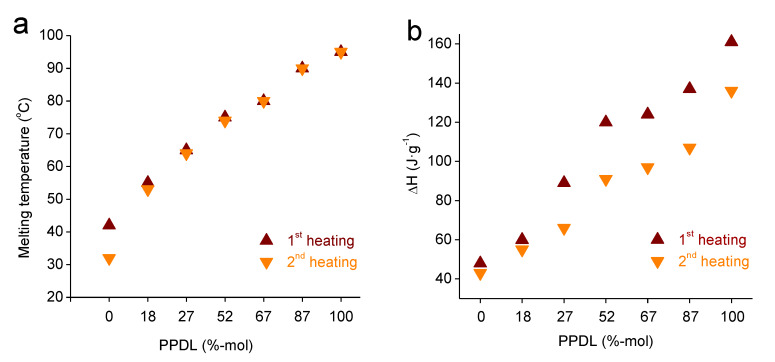
Melting temperatures (**a**) and enthalpies (**b**) of P(Gl_x_-*r*-PDL_y_) copolyesters as a function of their monomeric composition.

The DSC analysis of the P(Gl_x_-*r*-PDL_y_) copolyesters was performed by recording heating–cooling–reheating cycles over the −30 to 200 °C interval. The first heating and cooling traces are shown in Figure 1a and those produced at the second heating are depicted in Figure 1b. The heating traces recorded for PGl and PPDL displayed melting peaks corresponding to *T*_m_ at 42 and 95 °C, respectively, according to what is recurrently reported for these poly(macrolactone)s [18]. It was noticed, however, that the signal recorded for PGl was broader and displayed an associated enthalpy much lower than PPDL. Such features indicate that PGl crystallized more defectively and in less extent than PPDL, as it is consistent with the isomerism present in this unsaturated polyester. What is really interesting is that P(Gl_x_-*r*-PDL_y_) copolyesters were crystalline for every comonomeric content and that showed linearly increasing melting and enthalpy temperatures over the whole composition range as their content in PDL increased (Figure 2a). A similar trend was observed for the melting of the copolyesters recorded at the second heating with *T*_m_ values very close to those registered in the first heating run. These DSC results strongly suggest that Gl and PDL units must be isomorphic at crystallization, a distinguishing property of P(Glx-r-PDLy) copolyesters that deserves further insight. Such a study is currently under course to be published in a forthcoming paper.

### 3.2. Grafting of P(Gl_x_-r-PDL_y_) Copolyesters with Glutamic Acid Units: Synthesis of P[(Gl_x_-r-PDL_y_)–g–(LGlu)_z_] Copolymers

The presence of double bonds in the P(Gl_x_-*r*-PDL_y_) copolyesters made them suitable for the preparation of graft copolymers. As it is depicted in Scheme 1, amino functionalities were firstly inserted in the Gl units by thiol-ene reaction with 2-(Boc-amino)ethanethiol (BAET) followed by treatment with TFA to remove the Boc-protecting group. This method has been before proven to be effective for the preparation of multiaminated PGl used as macroinitiator for grafting with other amino acids by NCA ROP [21,27]. The evolution of the addition reaction was followed by ^1^H NMR that allowed determining the coupling efficiency of BAET, which was found to be 90% and 50% for P[(Gl_13_-*r*-PDL_87_) and P[(Gl_48_-*r*-PDL_52_), respectively (Figure S5 in Supplementary Materials).

**Figure 3:**
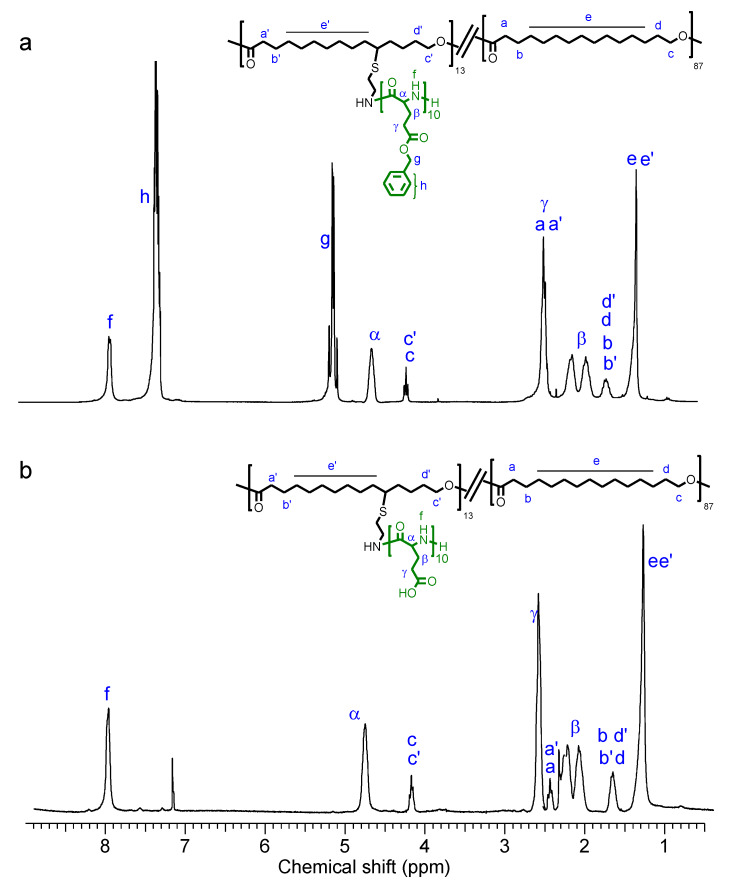
^1^H NMR (CDCl_3_/trifluoroacetic acid (TFA)) spectra of (**a**) P[(Gl_13_-*r*-PDL_87_)–*g*–(BLG)_10_] and (**b**) P[(Gl_13_-*r*-PDL_87_)–*g*–(LGA)_10_] copolymers.

The aminofunctionalized P[(Gl-NH_2_)_x_-*r*-PDL_y_] copolyesters were then grafted by ROP of the BLG-NCA. Amino groups initiated the grafting reaction with an amino conversion close to 100%. The GPC analysis of the grafted copolymers provided chromatograms consisting essentially in a single peak (other peaks appearing at longer elution times are due to salts added to the eluent, Figure S6 in Supplementary Materials) that confirmed the achievement of the grafting reaction and the absence of free homopolypeptide that could have been generated without the concourse of the amino groups. After acidic treatment of P[(Gl_x_-*r*-PDL_y_)–*g*–(BLG)_z_] with a TFA/HBr mixture, the COOH functionality of glutamic acid was readily regenerated to render P[(Gl_x_-*r*-PDL_y_)–*g*–(LGlu)_z_] copolymers which were then duly characterized by NMR. For illustrative purposes, the ^1^H NMR spectra of P[(Gl_13_-*r*-PDL_87_)–*g*–(BLG)_10_] and P[(Gl_13_-*r*-PDL_87_)–*g*–(LGA)_10_] are shown in Figure 3, and those registered from P[(Gl_48_-*r*-PDL_52_)–*g*–(BLG)_2_] and P[(Gl_48_-*r*-PDL_52_)–*g*–(LGA)_2_] are included in the SI file as Figure S7. Area comparison of the 5.15 ppm signal arising from BLG-CH_2_– with any of the signals characteristic of the copolyester (Figure 3a) demonstrated the success attained in the grafting reaction and allowed a precise estimation of the average length of the polypeptide chains grafted on the polyester. Additionally, the total absence of aromatic signals in the spectra of the deprotected copolymers, which is indicated by the disappearance of the 7.4 ppm signal, was taken as demonstrative that COOH groups had been fully recovered (Figure 3b). Compositions, yields, and average molecular weights of the grafted copolymers are collected in Table 3.

### 3.3. Thermal Properties and Structure of P[(Gl_x_-r-PDL_y_)–g–(LGlu)_z_] Copolymers

Thermal decomposition and transition temperatures of P[(Gl_x_-*r*-PDL_y_)–*g*–(LGlu)_z_] copolymers were measured by TGA and DSC, respectively. They are collected in Table 4. The TGA traces of the grafted copolymers as well as their respective derivative curves are displayed in Figure S8 of the SI file. A comparison of TGA results with those obtained for the P(Gl_x_-*r*-PDL_y_) copolyesters (Table 2) evidences that thermal stability underwent a notable decrease upon grafting. Furthermore, the decomposition process became more complex, and the amount of residue left after heating at 600 °C was noticeably higher, in particular, as far as deprotected copolymers are concerned. These results are in line with those results previously obtained for other amino acid-grafted polymacrolactones [21,27] and ratify the deleterious effect that the insertion of the polypeptide chains exerts on the thermal stability of the original copolyester.

**Figure 4:**
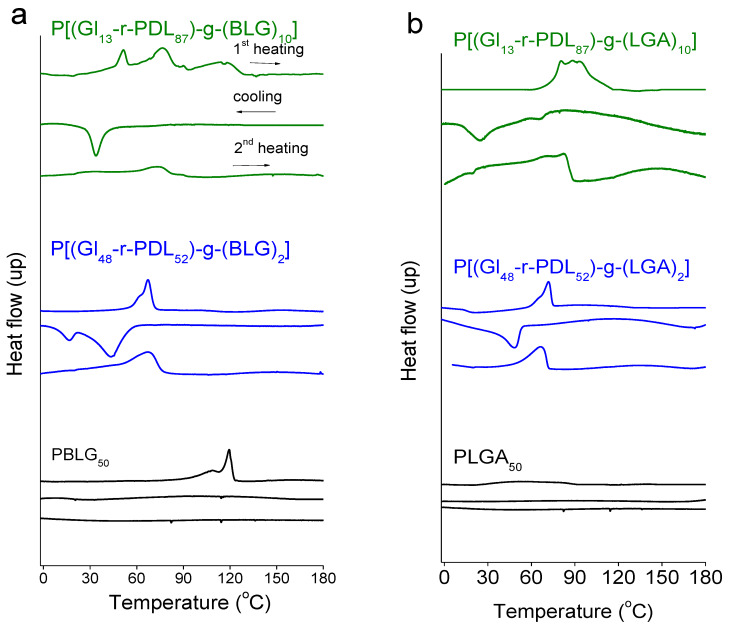
Heating and cooling DSC traces of P[(Gl_x_-*r*-PDL_y_)–*g*–(LGlu)_z_] copolymers.

The heating, cooling, and reheating DSC traces recorded from the P[(Gl_x_-*r*-PDL_y_)–*g*–(LGlu)_z_] copolymers together with those of the parent polypeptides are shown in Figure 4. Flat traces characteristic of amorphous material were invariably produced by PLGA, whereas the first heating trace of PBLG exhibited a sharp endothermal peak that is attributed in the literature to a nonreversible transition involving the rearrangement of the 18/5 to the 7/2 helical conformation [28,29,30]. DSC traces recorded from the copolymers showed, in the four cases, heat exchange peaks characteristic of melting, which are demonstrative of the strong propensity of polymacrolactones to crystallize. In fact, single or multiple endothermic peaks within the 50–90 °C range attributable to melting of PDL/Gl sequences, differing in length or/and monomeric composition, were observed on the first heating traces of the copolymers. The cooling traces showed crystallization exotherms at different supercooling degrees, and their corresponding melting peaks were recovered on the second heating traces. This thermal-crystallization behavior is in agreement with that observed for similar copolyesters obtained by ROP catalyzed by pyridylamidozinc(II) complex [22]. The broad endotherm that is observed exclusively on the first heating trace of P[(Gl_13_-*r*-PDL_87_)–*g*–(BLG)_10_] over the 115–120 °C range is attributed to the helical transition undergone by the (BLG)_10_ side chains. The inobservance of this transition in the other copolymers is according to expectations given the short number or/and charged state of the glutamic acid units in such cases.

**Figure 5:**
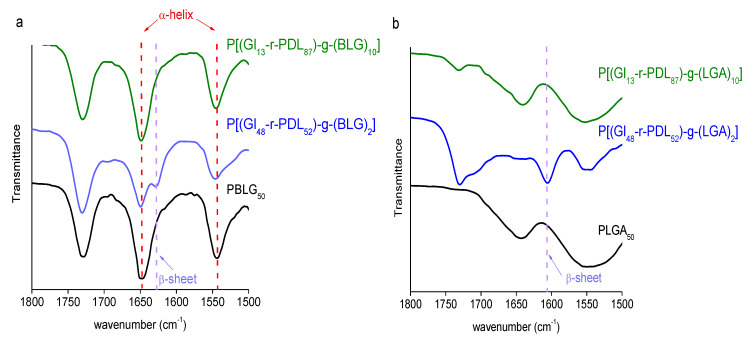
FTIR spectra of P[(Gl_x_-*r*-PDL_y_)–*g*–(LGlu)_z_] copolymers and reference polypeptides poly(γ-benzyl L-glutamate) (PBLG) and poly(L-glutamic acid) (PLGA).

The arrangement adopted by the polypeptide counterpart in P[(Gl_x_-*r*-PDL_y_)–*g*–(LGlu)_z_] copolymers was examined in detail by FTIR using homopolypeptides poly(γ-benzyl L-glutamate) (PBLG) and poly(L-glutamic acid) (PLGA) as reference. The 1800–1500 cm^−1^ region of the infrared spectra recorded from samples in the powder form is shown in Figure 5. The amide I and amide II bands appearing with strong intensity at 1655 and 1550 cm^−1^ on the spectra of both P[(Gl_13_-*r*-PDL_87_)–*g*–(BLG)_10_] and PBLG_50_, respectively, as well as the absence of absorption around 1630 cm^−1^, are solid indications of the arrangement of the (BLG)_10_ side chain in α-helix conformation [31,32,33]. On the contrary, the spectra produced by P[(Gl_48_-*r*-PDL_52_)–*g*–(BLG)_2_] show a conspicuous peak at 1630 cm^−1^ consistent with the presence of a considerable amount of the (BLG)_2_ in β-form. The spectra of the unprotected copolymers showed broad amide bands more according with the polypeptide in a disordered state although the band at 1620 cm^−1^ observed in the spectrum of P[(Gl_48_-*r*-PDL_52_)–*g*–(LGA)_2_] suggests the presence of some β-form in this copolymer. These FTIR results are in good agreement with that is commonly accepted for the conformational properties of polypeptides, i.e., the α-helical conformation is favored by longer amino acid sequences but becomes incompatible with the ionically charged form [34,35]. The effect of temperature on the polypeptide conformation was also examined for the P[(Gl_13_-*r*-PDL_87_)–*g*–(BLG)_10_] and P[(Gl_48_-*r*-PDL_52_)–*g*–(BLG)_2_] copolymers within the 20–200 °C range (spectra are reproduced in Figure S9 of SI). Neither of the two cases showed apparent spectral changes indicative of α-helix/β-sheet interconversion or helical disruption into the random coil state over the assayed temperature interval. This is in full agreement with the high thermal stability of the α and β forms of polypeptides, which are known to resist temperatures up to well above 200 °C [36].

**Figure 6:**
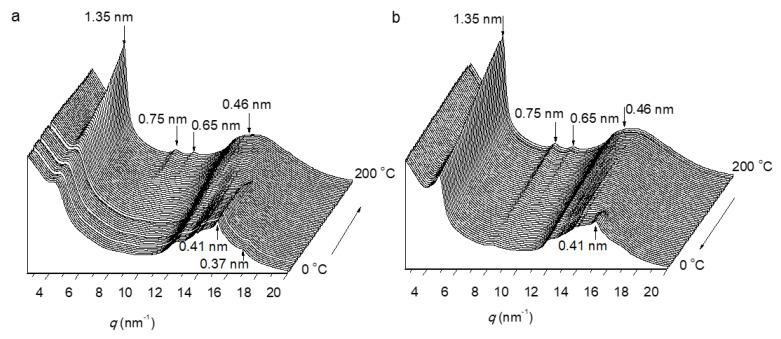
Evolution of the XRD profiles recorded at heating (**a**) and cooling (**b**) from P[(Gl_13_-*r*-PDL_87_)–*g*–(BLG)_10_] copolymer over the 0–200 °C range.

X-ray diffraction (XRD) of the hybrid copolymers in the solid state afforded complementary information in support of the results obtained by both DSC and FTIR. XRD profiles of the P[(Gl_13_-*r*-PDL_87_)–*g*–(BLG)_10_] copolymer recorded at real time at temperatures varying over the 0–200 °C range are shown in Figure 6. The 0.41 and 0.37 nm peaks present in the low temperature profiles registered at both heating and cooling are interpreted to arise from the crystal structure of the P(Gl_x_-*r*-PDL_y_) copolyesters. According to DSC results, these peaks disappeared at temperatures above melting, i.e., ~75 °C to be replaced by a broad peak at 0.46 nm arising from the disordered state. Additionally, a set of peaks corresponding to lattice spacings roughly related by the 1:√3:2 ratio, (i.e., 1.35, 0.75, and 0.65 nm) was observed. As it has been previously reported on several occasions [37,38,39], such diffraction pattern is indicative of the occurrence of a two-dimensional hexagonal packing of PBLG α-helices with a diameter of approximately 1.5 nm. A detailed inspection of the variation of such pattern with temperature revealed that all three peaks were intensified at high temperatures to practically disappear upon cooling below the temperature at which the polyester crystallized. This behavior, which has been observed before for block copolymers made of PPDL and PBLG [36,37], suggests that melting of the polyester phase favored the building of the polypeptide columnar phase. Results obtained in similar wide/small angle X-ray scattering (WAXS/SAXS) analyses carried out on P[(Gl_48_-*r*-PDL_52_)–*g*–(BLG)_2_] are provided in Figure S10 of SI. In this case, the diffraction peaks characteristic of the crystallized polyester observed at low temperatures were not recovered after cooling and no diffraction peaks characteristic of columnar phase were detected. On the other hand, the discrete scattering registered for the deprotected copolymers was limited to the 0.41 and 0.37 nm peaks characteristic of polyester melting. All other signals detected in these profiles were very weak and of difficult interpretation (Figure S11 in SI).

### 3.4. Self-Assembly of P[(Gl_x_-r-PDL_y_)–g–(LGlu)_z_] Copolymers: DOX Loading and Releasing

**Figure 7:**
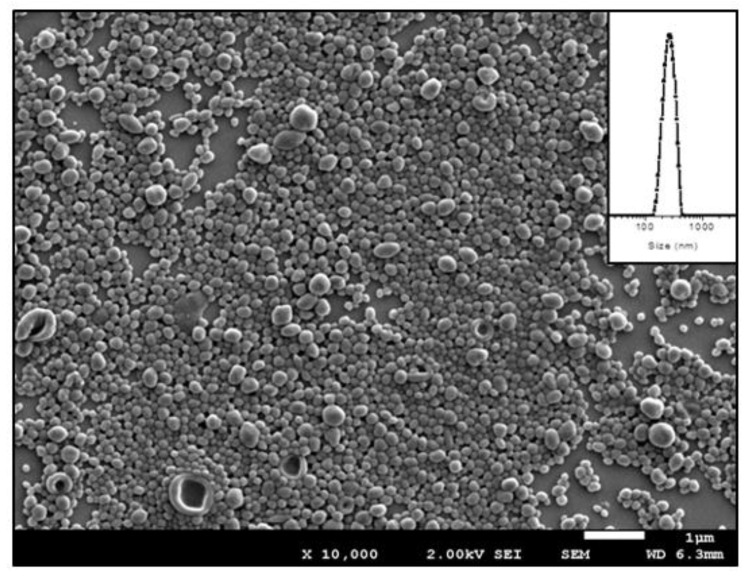
SEM image of nanoparticles made of P[(Gl_13_-*r*-PDL_87_)–*g*–(BLG)_10_] copolymer. Inset: dynamic light scattering studies (DLS) profile recorded from the particle suspension. Additional SEM images are available in Figure S12 of SI.

The chemical form in which the amino acid units are found in the P[(Gl_x_-*r*-PDL_y_)–*g*–(LGlu)_z_], i.e., with the carboxyl group free or in the ester form, determined their solubility and consequently, the procedure suitable for promoting their self-assembly into nanometric entities. Thus, the emulsion-solvent evaporation method was applied to the nonwater-soluble P[(Gl_13_-*r*-PDL_87_)–*g*–(BLG)_10_] copolymer to obtain spherical nanoparticles of average diameter around 250 nm displaying an acceptable polydispersity and a zeta potential of −3.75 mV. The SEM analysis of these nanoparticles revealed that they have a nearly round shape and are well delineated without showing apparent aggregation (Figure 7 and Figure S12 of SI).

Deprotection of the benzyl carboxylate groups of P[(Gl_13_-*r*-PDL_87_)–*g*–(BLG)_10_] led to the strongly amphiphilic and water-soluble P[(Gl_13_-*r*-PDL_87_)–*g*–(LGA)_10_] copolymer. The emulsion-solvent evaporation method successfully used with the protected copolymer was not applicable after deprotection because the carboxylic copolymer is not soluble in the usual volatile solvents required for preparing the organic solution. Conversely, when P[(Gl_13_-*r*-PDL_87_)–*g*–(LGA)_10_] was dissolved in water at concentrations above 0.5 mg·mL^−1^, it became spontaneously self-organized to form micelle-like objects as it was revealed by the DLS analysis. The critical micelle concentration (cmc) of P[(Gl_13_-*r*-PDL_87_)–*g*–(LGA)_10_] measured by DLS was 0.15 mg·mL^−1^ (Figure S13 in SI). The micelle size increased with copolymer concentration with average diameter values ranging between 200 and 600 nm to become bimodal at 3.0 mg·mL^−1^ (Figure S13 of SI). The Z-potential of these micelles was negative with a value around −34 mV as it should be expected for a localization of the carboxylate groups preferably on the particle surface.

**Figure 8:**
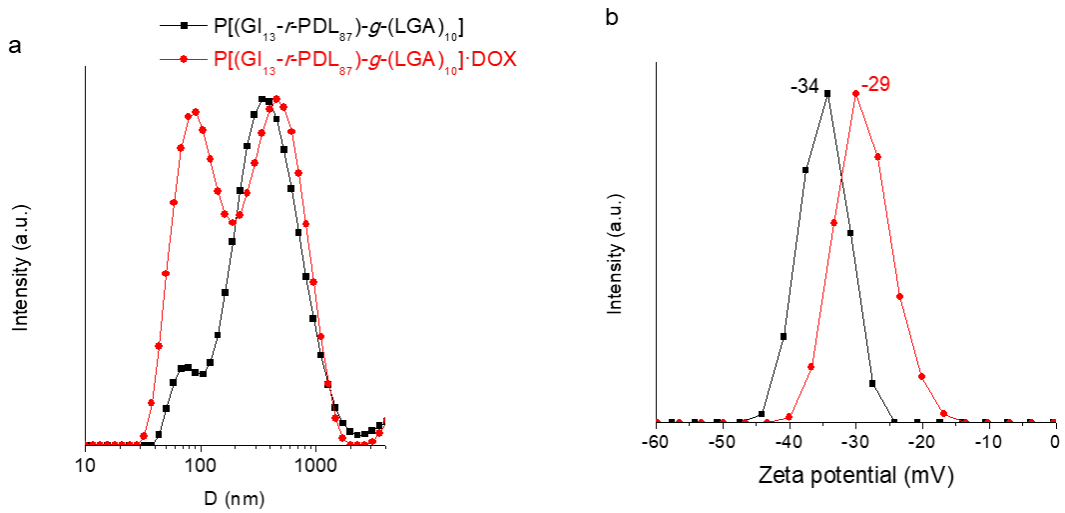
DLS profiles (**a**) and zeta potential (**b**) of the P[(Gl_13_-*r*-PDL_87_)–*g*–(LGA)_10_] copolymer and the P[(Gl_13_-*r*-PDL_87_)–*g*–(LGA)_10_]·doxorubicin (DOX) conjugate.

DOX is a well-known amphiphilic drug that is commonly used in cancer therapy [40]. The amino group attached to the sugar moiety of the DOX molecule becomes positively charged at pH below 5 (Figure S14 in SI). DOX hydrochloride (DOX·HCl) has been used in a large number of occasions to test the drug-loading capacity of nanoparticles made of carboxylic polymers that are able to interact electrostatically with the NH_3_^+^ group provided that they are located at the particle surface [41,42,43,44,45,46,47]. To assess the potential of P[(Gl_13_-*r*-PDL_87_)–*g*–(LGA)_10_] as nanocarrier, this copolymer was mixed with DOX·HCl in water at different LGA:drug molar ratios (1:0.4, 1:0.15, and 1:0.07), and the resulting DOX-loaded micelles were examined by DLS. The results found for 1.25 and 3 mg·mL^−1^ copolymer concentrations with an LGA:drug ratio of 1:0.4 are shown in Figure 8. The copolymer particles without DOX showed an essentially unimodal distribution of sizes with an average diameter of 250 nm. On the contrary, the DLS of the DOX-loaded particles revealed a bimodal distribution with average diameters of about 80–400 nm. The negative zeta potential of the particles decreased upon loading as a logical consequence of the partial neutralization of the negative charge that takes place by electrostatic interaction with DOX.

**Figure 9:**
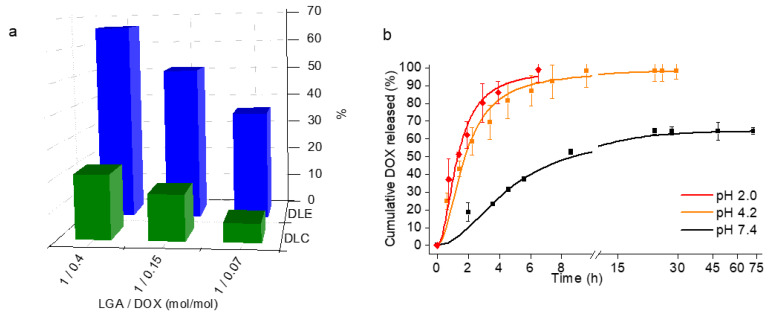
(**a**) Drug loading efficiency (DLE) and drug loading content (DLC) of P[(Gl_13_-*r*-PDL_87_)–*g*–(LGA)_10_]·DOX at different l-glutamic acid (LGA)/DOX ratios. (**b**) Cumulative releasing profiles of DOX at the three indicated pHs. The data presented are mean ± SD (n = 3).

The suitability of P[(Gl_13_-*r*-PDL_87_)–*g*–(LGA)_10_] for DOX loading was assessed by estimating the drug-loading efficiency (DLE) and drug-loading content (DLC) for the three LGA:drug molar ratios tested in this study. The bar plot in Figure 9a shows the DLE and DLC values estimated for the three cases. Both parameters increased with the increasing amount of added DOX to attain values of 66% and 21%, respectively, for the LGA:DOX = 1:0.4, which was the lowest ratio assayed. This composition was chosen for evaluating the ability of P[(Gl_13_-*r*-PDL_87_)–*g*–(LGA)_10_]·DOX to deliver the drug as well as to assess the response of the system to pH changes. The DOX-releasing profiles obtained upon incubation of this conjugate at pH 2.0, 4.2, and 7.4 are compared in Figure 9b. In both cases, DOX was delivered following an asymptotic function of time without any detected burst confirming that all the loaded DOX was chemically attached to the copolymer. The maximum amount of delivered DOX under neutral conditions was about 60%, and it was attained after one day of incubation. On the other hand, more than 95% of DOX was released in 10 and 6 h when the loaded particles were incubated at pH 4.2 and 2.0, respectively. The observed differences must be attributed to the notable decrease in the ionization degree of the LGA moieties that is produced at acidic pH with the subsequent disruption of the copolymer–drug electrostatic interactions. 

## 4. Conclusions

Random copolyesters P(Gl_x_-*r*-PDL_y_) made of two macrolactones, i.e., globalide (Gl) and pentadecalactone (PDL), and covering the whole range of compositions as well as the homopolyesters PGl and PPDL were successfully prepared by enzymatic ROP. The DSC study demonstrated that the copolyesters were crystalline and able to recrystallize from the melt. Crystallinity and density of double bonds in these copolyesters were tuned by composition. Amino acid grafting of P(Gl_x_-*r*-PDL_y_) was satisfactorily performed by ROP of BLG-NCA initiated by amino functions previously inserted in the Gl units of the copolyester. The benzyl-protecting group could be then readily removed to produce strongly amphiphilic copolyesters bearing free carboxylate groups in the grafting side chains. Both the amount of grafted Gl units and the average length of the grafting polyglutamate side chains could be accurately controlled so that copolymers displaying different water solubility were prepared. Neutral nonwater-soluble copolymers were able to self-assemble in spherical nanoparticles with an average diameter of 200–300 nm. These nanoparticles based on graft copolyester are unique in containing a crystalline highly hydrophobic core covered by a shell of polypeptide in α-helical conformation. On the other hand, the water-soluble graft copolyesters bearing ionized glutamic acid side chains produced micelles that were able to load fair amounts of DOX with a high capturing efficiency. Electrostatic ammonium–carboxylate interactions were responsible for the good loading capacity exhibited by the graft copolymer and also for the remarkable changes in the drug delivery profile displayed by the DOX-loaded micelles when incubated in aqueous medium at different pHs.

## Figures and Tables

**Table 1:** Yield, composition, and molecular weight of the P(Gl_x_-*r*-PDL_y_) copolyesters.

Polyester ^a^	Yield (%)	Composition ^b^ [Gl]/[PDL] (mol/mol)		*M*_n_^c^ (g·mol^−1^)
		Feed	Copolymer	
PGl	70	100/0	100/0	9700
P(Gl_82_-*r*-PDL_18_)	80	90/10	82/18	10,500
P(Gl_73_-*r*-PDL_27_)	75	70/30	73/27	9450
P(Gl_48_-*r*-PDL_52_)	88	50/50	48/52	11,900
P(Gl_33_-*r*-PDL_67_)	80	30/70	33/67	10,650
P(Gl_13_-*r*-PDL_87_)	90	10/90	13/87	11,760
PPDL	90	0/100	0/100	12,300

^a^ Subscripts x and y indicate the %-mole composition of the copolymer in globalide (Gl) and pentadecalactone (PDL) units, respectively; ^b^ %-mole composition of the feed and the copolymer determined by ^1^H NMR; and ^c^ number-average molecular weight of the copolymer as determined by end-group ^1^H NMR analysis.

**Table 2:** Thermal properties of P(Gl_x_-*r*-PDL_y_) copolyesters.

Copolyester		TGA ^a^		DSC ^b^					
				First Heating		Cooling		Second Heating	
	*T*_d_*^a^* (°C)	*^max^T*_d_ (°C)	*R*_w_ (%)	*T*_m_ (°C)	Δ*H_m_* (J·g^−1^)	*T*_c_ (°C)	Δ*H*_c_ (J·g^−1^)	*T*_m_ (°C)	Δ*H*_m_ (J·g^−1^)
PGl	349	419, 466	4	42	48	17	−35	27	43
P(Gl_82_-*r*-PDL_18_)	376	417, 466	2	54	60	38	−40	53	55
P(Gl_73_-*r*-PDL_27_)	387	420, 469	1	65	89	50	−46	66	66
P(Gl_48_-*r*-PDL_52_)	378	423, 470	1	74	120	57	−76	74	91
P(Gl_33_-*r*-PDL_67_)	374	431, 469	1	81	124	65	−93	80	97
P(Gl_13_-*r*-PDL_87_)	381	426, 470	1	90	137	74	−101	90	107
PPDL	396	427, 472	1	95	161	81	−130	95	136

^a^ Onset for 5% weight loss (*T*_d_) and maximum rate (*^max^T*_d_) thermal decomposition temperatures measured by TGA under inert atmosphere. *R*_w_*:* weight (%) remaining after heating at 600 °C and ^b^ melting (*T*_m_ and Δ*H*_m_) and crystallization (*T*_c_ and Δ*H*_c_) temperatures and enthalpies measured by differential scanning calorimetry (DSC).

**Table 3:** P[(Gl_x_-*r*-PDL_y_)–*g*–(LGlu)_z_] copolymers: composition and molecular weight.

Copolymer ^a^	Gl-BAET ^b^ (%)	Copolymer ^c^ Gl/PDL/LGlu	Side Chain Length ^d^ (LGlu Units)	Yield ^e^ (%)	*M*_n_^f^ (g·mol^−1^)
P[(Gl_13_-*r*-PDL_87_)–*g*–(BLG)_10_]	90	6/38/56	10	93	27,700
P[(Gl_48_-*r*-PDL_52_)–*g*–(BLG)_2_]	50	32/36/32	2	85	16,700
P[(Gl_13_-*r*-PDL_87_)–*g*–(LGA)_10_]	90	6/38/56	10	70	21,400
P[(Gl_48_-*r*-PDL_52_)–*g*–(LGA)_2_]	50	32/36/32	2	74	15,100

^a^ Subscripts for Gl (including both modified and unmodified units) and PDL stand for their molar percentages in the copolymer. Subscripts for γ-benzyl-L-glutamate (BLG) and l-glutamic acid (LGA) are the number average of such units in the grafting side chains; ^b^ molar percentage of aminofunctionalized Gl units estimated by ^1^H NMR, calculated by comparing areas of peaks arising from the double bond in PGl with the peak areas corresponding to the methylene group adjacent to ester group; ^c^ copolymer composition (%-mole) in Gl (both modified and unmodified units), PDL and LGlu (BLG or LGA) units; ^d^ average number of L-Glu (either BLG or LGA) units in the side chains of the grafted copolyesters; ^e^ yield of the grafting reaction; and ^f^ number-average molecular weight of the copolymer determined by ^1^H NMR.

**Table 4:** Thermal properties of P[(Gl_x_-*r*-PDL_y_)–*g*–(LGlu)_z_] copolymers.

Copolymer	TGA ^a^			DSC ^b^							
				First Heating				Cooling		Second Heating	
	*T*_d_ °C	*^max^T*_d_ °C	*R*_w_ %	*T*_m_ °C	Δ*H* J·g^−1^	*T*_LC_^c^ °C	Δ*H* J·g^−1^	*T*_c_ °C	Δ*H* J·g^−1^	*T*_m_ °C	Δ*H* J·g^−1^
P[(Gl_13_-*r*-PDL_87_)–*g*–(BLG)_10_]	235	230–280	18	52,76	4	115–120	2	34	−6	75	6
P[(Gl_48_-*r*-PDL_52_)–*g*–(BLG)_2_]	220	290–320	12	68	21	-	-	43	−13	68	16
P[(Gl_13_-*r*-PDL_87_)–*g*–(LGA)_10_]	285	310–450	42	80–90	9	-	-	58	−0.5	83	1
P[(Gl_48_-*r*-PDL_52_)–*g*–(LGA)_2_]	215	270–400	29	72	18	-	-	48	−9	67	11

^a^ Onset temperature for 5% of weight loss (*T*_d_), maximum rate decomposition temperature (*^max^T*_d_) and remaining weight (*R*_w_) after heating at 600 °C; ^b^ glass transition (*T*_g_), melting (*T*_m_ and **Δ***H*_m_), and crystallization (*T*_c_ and **Δ***H*_c_) temperatures and enthalpies measured by DSC; and ^c^ *T*_LC_ is the temperature for the structural transition undergone by the polypeptide hexagonal phase.

## Associated Information

^1^H and ^13^C NMR spectra of PGl_x_-*r*-PDL_y_ copolyesters. ^13^C NMR spectra of Gl and PGl. ^1^H NMR spectra and GPC chromatograms of P[(Gl_x_-*r*-PDL_y_)–*g*–(LGlu)_z_] copolymers. TGA plots and derivative curves of P[(Gl_x_-*r*-PDL_y_)] copolyesters and grafted copolymers. FTIR spectra of the P[(Gl_x_-*r*-PDL_y_)–*g*–(BLG)_z_] graft copolymers at different temperatures. X-ray diffraction profiles of the P[(Gl_48_-*r*-PDL_52_)–*g*–(BLG)_2_] graft-copolymer. Additional SEM images of NPs made of the P[(Gl_13_-*r*-PDL_87_)–*g*–(BLG)_10_] copolymer. DLS profiles and critical micelle concentration of micelles-like objects made of P[(Gl_13_-*r*-PDL_87_)–*g*–(LGA)_10_]. Chemical structure of DOX·HCl.

## Supplementary Materials

The following are available online at https://www.mdpi.com/2073-4360/12/4/995/s1, Figure S1: ^1^H NMR (a) and ^13^C NMR (b) of the P(Gl_13_-*r*-PDL_87_) copolyester registered in CDCl_3_; Figure S2: ^1^H NMR (CDCl_3_) of the P(Gl_x_-*r*-PDL_y_) copolyesters series; Figure S3: ^13^C NMR spectra of Gl and PGl highlighting the characteristic peaks of the different isomers used for quantification; Figure S4: TGA traces (a) and derivative curves (b) of the P(Gl_x_-*r*-PDL_y_) copolyesters; Figure S5: ^1^H NMR (CDCl_3_) spectra of the P[(Gl-BAET)_13_-*r*-PDL_87_] (a) and P[(Gl-NH_2_)_13_*-r*-PDL_87_] (b); Figure S6: GPC curves of the P[(Gl_x_-*r*-PDL_y_)-*g*-(LGlu)_z_] copolymers. Peaks observed at elution times longer than 25 min (framed area) are due to salts added to the running solvent; Figure S7: ^1^H NMR (CDCl_3_/TFA) spectra of the P[(Gl_48_-*r*-PDL_52_)-*g*-(BLG)_2_] (a), and P[(Gl_48_-*r*-PDL_52_)-*g*-(LGA)_2_] (b); Figure S8: TGA traces (a,b) and derivative curves (a’,b’) of the P[(Gl_x_-*r*-PDL_y_)-*g*-(BLG)_z_] and P[(Gl_x_-*r*-PDL_y_)-*g*-(LGA)_z_] copolymers; Figure S9: 1800–1500 cm^−1^ region of FTIR spectra of P[(Gl_13_-*r*-PDL_87_)-*g*-(BLG)_10_] (a) and P[(Gl_48_-*r*-PDL_52_)-*g*-(BLG)_2_] (b) at different temperatures over the 20–200 °C range; Figure S10: evolution of the WAXS (a) and SAXS (b) profiles recorded from P[(Gl_48_-*r*-PDL_52_)-*g*-(BLG)_2_] copolymer at heating over the 10–200 °C range; Figure S11: evolution of the WAXS (a) and SAXS (b) profiles recorded from P[(Gl_48_-*r*-PDL_52_)-*g*-(LGA)_2_] copolymer at heating over the 0–200 °C range; Figure S12: SEM images of nanoparticles made of P[(Gl_13_-*r*-PDL_87_)-*g*-(BLG)_10_]; Figure S13: DLS profiles (a) and plot used for determining the critical concentration (b) of micelles made of P[(Gl_13_-*r*-PDL_87_)-*g*-(LGA)_10_]; Figure S14: chemical structure of DOX·HCl.
