# Supplementary material for: Copolymacrolactones Grafted with l-Glutamic Acid: Synthesis, Structure, and Nanocarrier Properties
Source: Polymers (Basel). 2020 Apr 24;12(4):995. doi: 10.3390/polym12040995 (PMC7240413; doi:10.3390/polym12040995)
Supplement: Supplementary file 1 [file polymers-12-00995-s001.pdf]

Copolymacrolactones Grafted with L-Glutamic Acid: Synthesis, Structure, and Nanocarrier Properties

Ernesto Tinajero-Díaz, Antxon Martínez de Ilarduya and Sebastián Muñoz-Guerra*

Departament d´Enginyeria Química, Universitat Politècnica de Catalunya, ETSEIB, Diagonal 647, 08028, Barcelona, Spain; ernesto.tinajero@gmail.com (E.T.-D.); antxon.martinez.de.ilarduia@upc.edu (A.M.I.)

* Correspondence: sebastian.munoz@upc.edu

Figure S1. ^{1}H NMR (a) and ^{13}C NMR (b) of the P(Gl$_{13}$-*r*-PDL$_{87}$) copolyester registered in CDCl$_3$.

Figure S2. ^{1}H NMR (CDCl$_3$) of the P(Gl$_x$-*r*-PDL$_y$) copolyesters series.

Figure S3. ^{13}C NMR spectra of Gl and PGl highlighting the characteristic peaks of the different isomers used for quantification.

Figure S4. TGA traces (a) and derivative curves (b) of the P(Gl$_x$-*r*-PDL$_y$) copolyesters.

Figure S5. ^{1}H NMR (CDCl$_3$) spectra of the P[(Gl-BAET)$_{13}$-*r*-PDL$_{87}$] (a), and P[(Gl-NH$_2$)$_{13}$-*r*-PDL$_{87}$] (b).

Figure S6. GPC curves of the P[(Gl$_x$-*r*-PDL$_y$)-*g*-(LGlu)$_z$] copolymers. Peaks observed at elution times longer than 25 min (framed area) are due to salts added to the running solvent.

Figure S7. ^{1}H NMR (CDCl$_3$/TFA) spectra of the P[(Gl$_{48}$-*r*-PDL$_{52}$)-*g*-(BLG)$_2$] (a), and P[(Gl$_{48}$-*r*-PDL$_{52}$)-*g*-(LGA)$_2$] (b).

Figure S8. TGA traces (a, b) and derivative curves (a′, b′) of the P[(Gl$_x$-*r*-PDL$_y$)-*g*-(BLG)$_z$] and P[(Gl$_x$-*r*-PDL$_y$)-*g*-(LGA)$_z$] copolymers.

Figure S9. 1,800-1,500 cm^{-1} region of FTIR spectra of P[(Gl$_{13}$-*r*-PDL$_{87}$)-*g*-(BLG)$_{10}$] (a) and P[(Gl$_{48}$-*r*-PDL$_{52}$)-*g*-(BLG)$_2$] (b) at different temperatures over the 20-200 ºC range.

Figure S10. Evolution of the WAXS (a) and SAXS (b) profiles recorded from P[(Gl$_{48}$-*r*-PDL$_{52}$)-*g*-(BLG)$_2$] copolymer at heating over the 10-200 ºC range.

Figure S11. Evolution of the WAXS (a) and SAXS (b) profiles recorded from P[(Gl$_{48}$-*r*-PDL$_{52}$)-*g*-(LGA)$_2$] copolymer at heating over the 0-200 °C range.

Figure S12. SEM images of nanoparticles made of P[(Gl$_{13}$-*r*-PDL$_{87}$)-*g*-(BLG)$_{10}$].

Figure S13. DLS profiles (a) and plot used for determining the critical concentration (b) of micelles made of P[(Gl$_{13}$-*r*-PDL$_{87}$)-*g*-(LGA)$_{10}$].

Figure S14. Chemical structure of DOX·HCl.

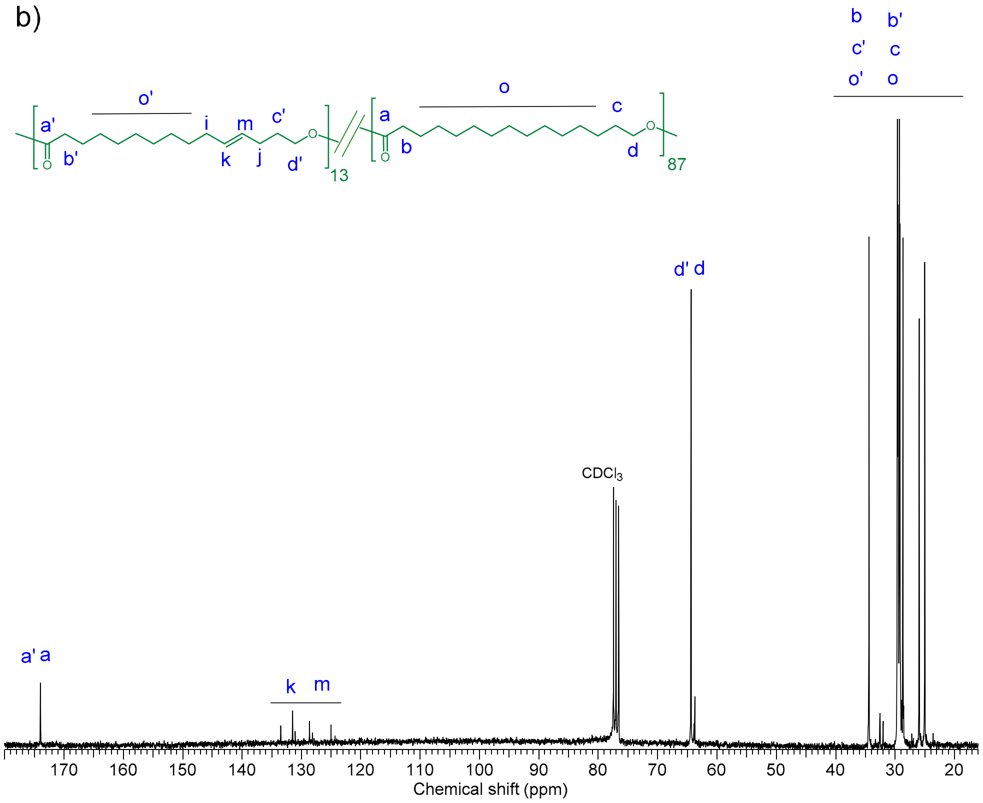

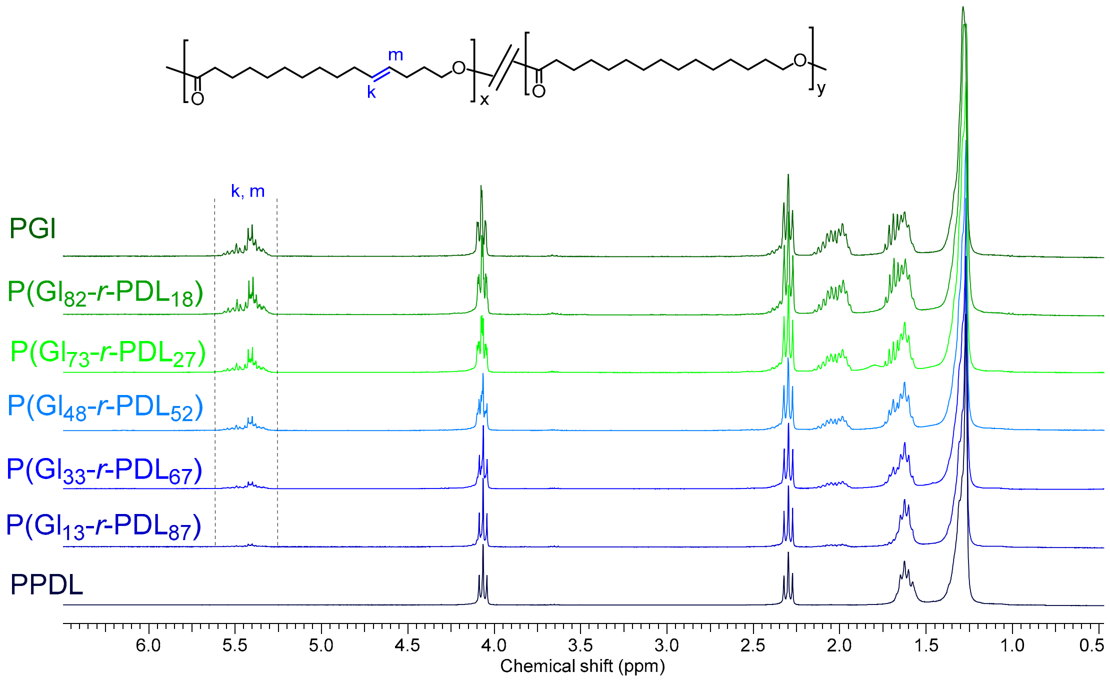

Figure S1. ^{1}H NMR (a) and ^{13}C NMR (b) of the P(Gl_{13}-*r*-PDL_{87}) copolyester registered in $CDCl_3$.

Figure S2. ^{1}H NMR ($CDCl_3$) of the P(Gl_x-*r*-PDL_y) copolyesters series.

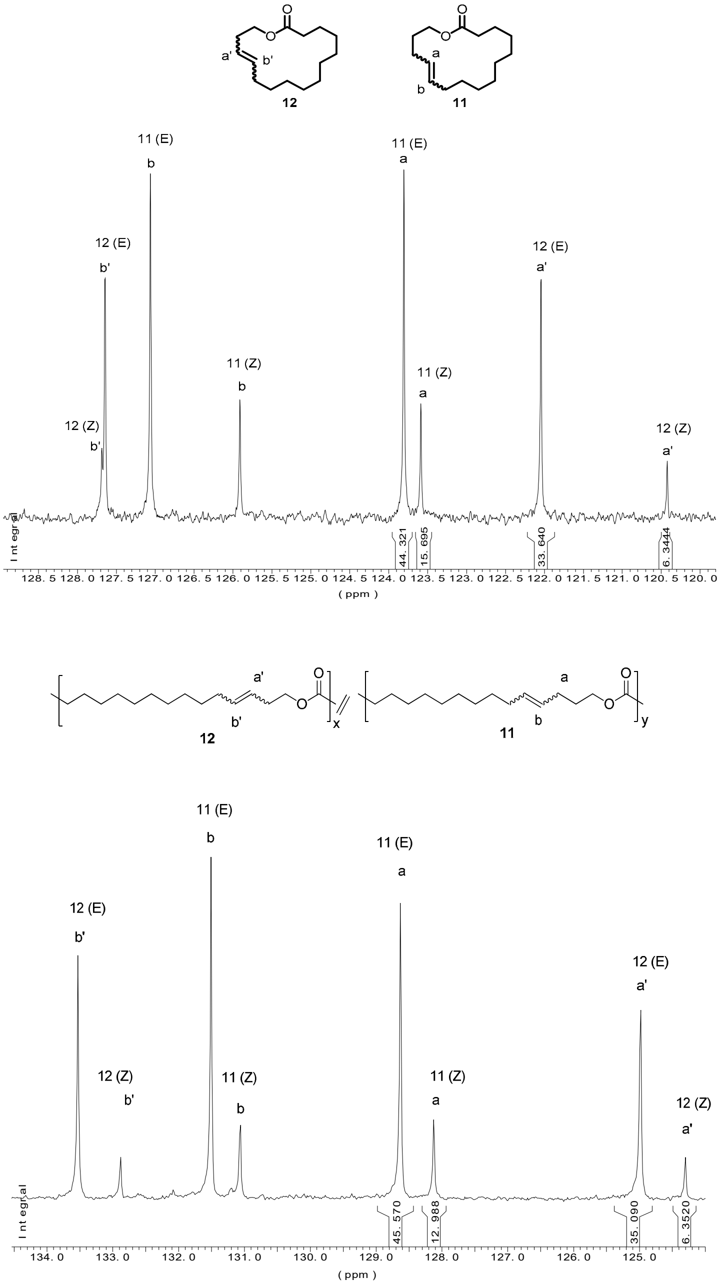

Sample	% mol-mass isomers			
	oxapentadecen-12-one		oxapentadecen-13-one	
	E	Z	E	Z
Gl	44.3	15.7	33.6	6.3
PGl	45.6	13.0	35.1	6.3

Figure S3. ^{13}C NMR ($CDCl_3$) spectra of Gl and PGl highlighting the characteristic peaks of the different isomers used for their quantification.

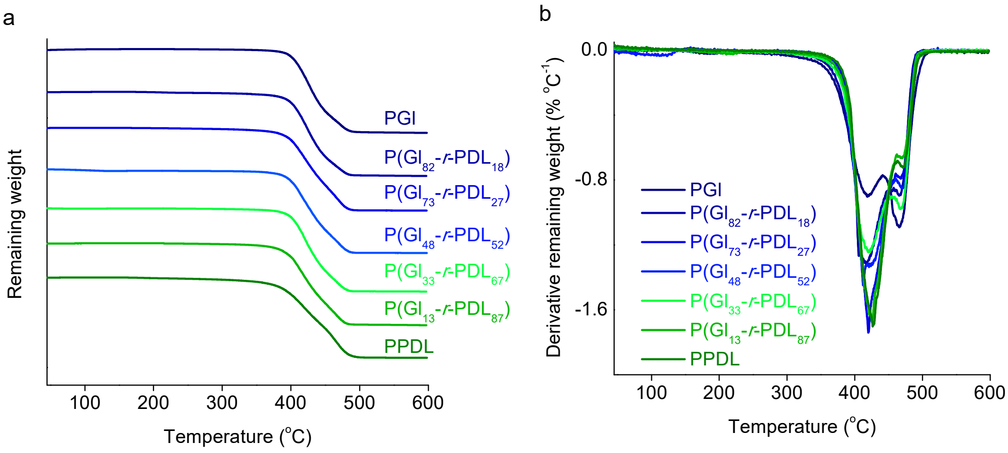

Figure S4. TGA traces (a) and derivative curves (b) of the P(Gl$_x$-*r*-PDL$_y$) copolyesters.

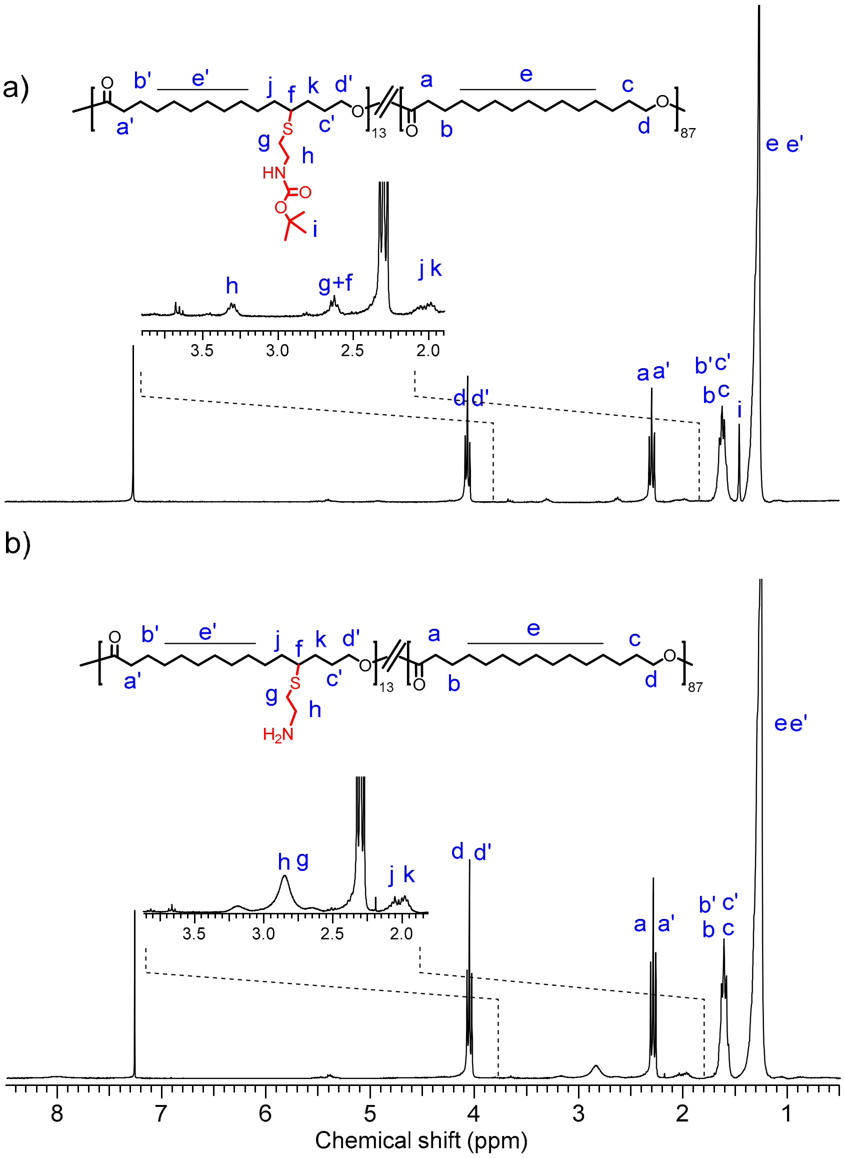

Figure S5. ^{1}H NMR ($CDCl_3$) spectra of the P[(Gl-BAET)$_{13}$-*r*-PDL$_{87}$] (a), and P[(Gl-NH$_2$)$_{13}$-*r*-PDL$_{87}$] (b).

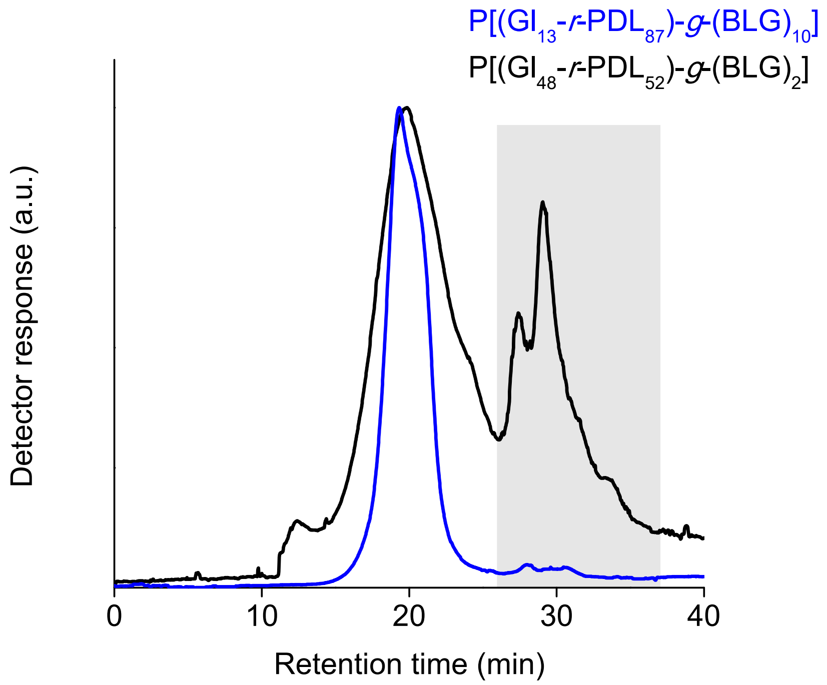

Figure S6. GPC curves of the P[(Gl$_x$-*r*-PDL$_y$)-*g*-(LGlu)$_z$] copolymers. Peaks observed at elution times longer than 25 min (framed area) are due to the sodium trifluoroacetate salts added to the running solvent.

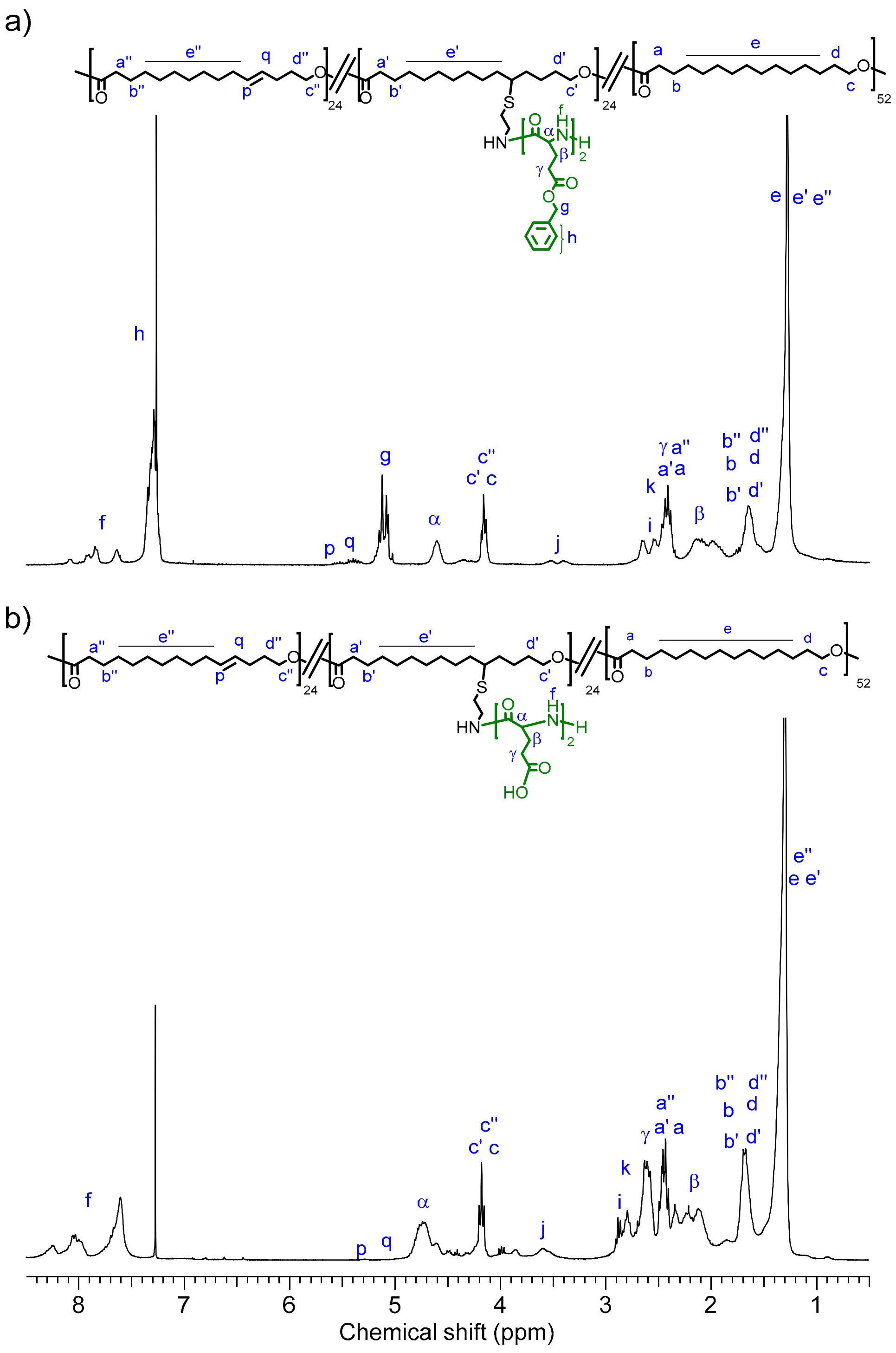

Figure S7. ^{1}H NMR ($CDCl_3$/TFA) spectra of the P[(Gl_{48}-*r*-PDL_{52})-*g*-$(BLG)_2$] (a), and P[(Gl_{48}-*r*-PDL_{52})-*g*-$(LGA)_2$] (b).

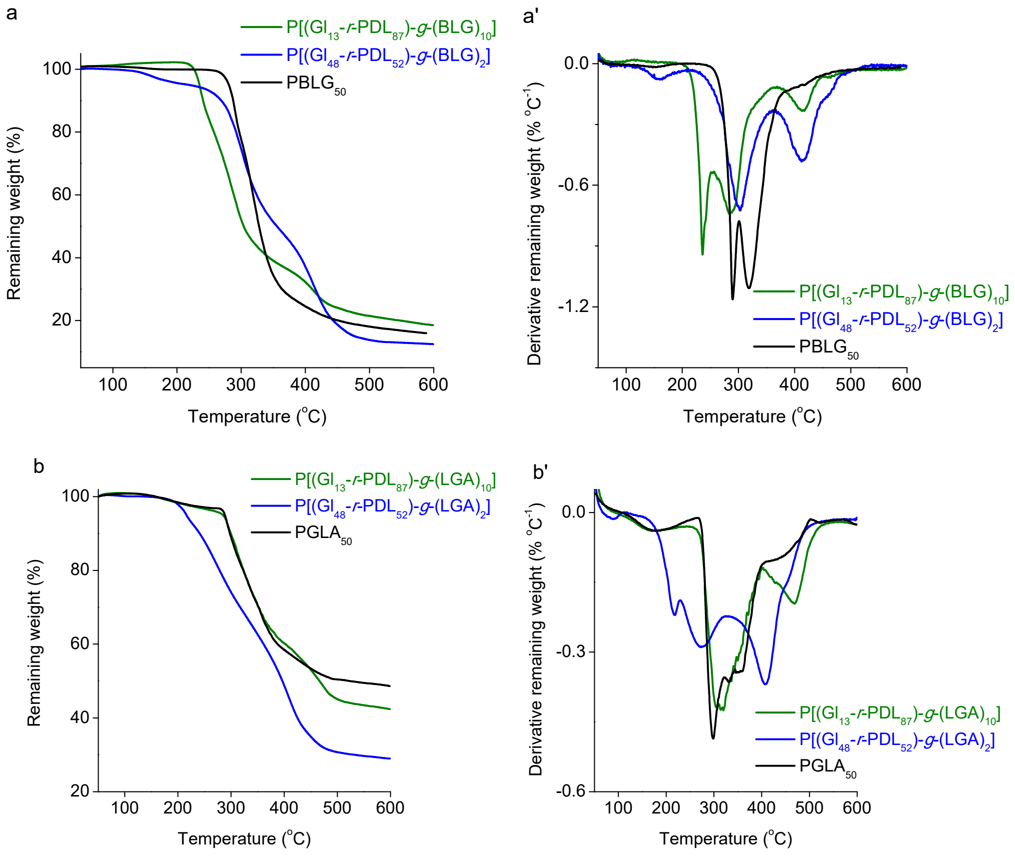

Figure S8. TGA traces (a, b) and derivative curves (a′, b′) of the P[(Gl$_x$-*r*-PDL$_y$)-*g*-(BLG)$_z$] and P[(Gl$_x$-*r*-PDL$_y$)-*g*-(LGA)$_z$] copolymers.

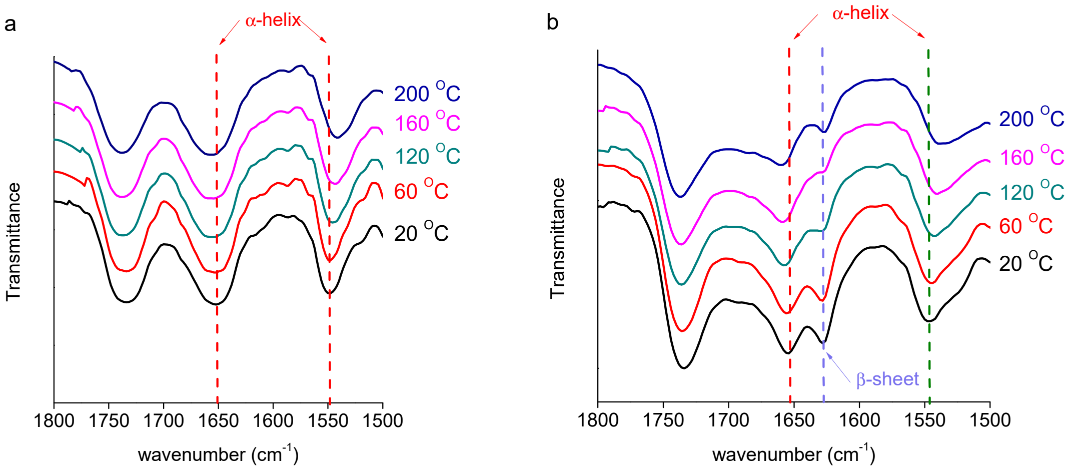

Figure S9. 1,800-1,500 cm^{-1} region of FTIR spectra of P[(Gl$_{13}$-*r*-PDL$_{87}$)-*g*-(BLG)$_{10}$] (a) and P[(Gl$_{48}$-*r*-PDL$_{52}$)-*g*-(BLG)$_2$] (b) at different temperatures over the 20-200 ºC range.

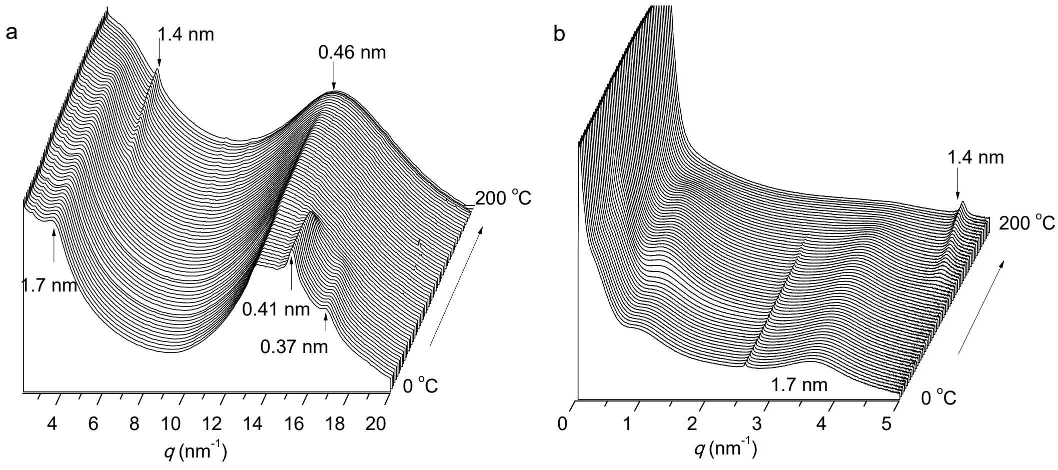

Figure S10. Evolution of the WAXS (a) and SAXS (b) profiles recorded from P[(Gl$_{48}$-*r*-PDL$_{52}$)-*g*-(BLG)$_2$] copolymer at heating over the 0-200 ºC range.

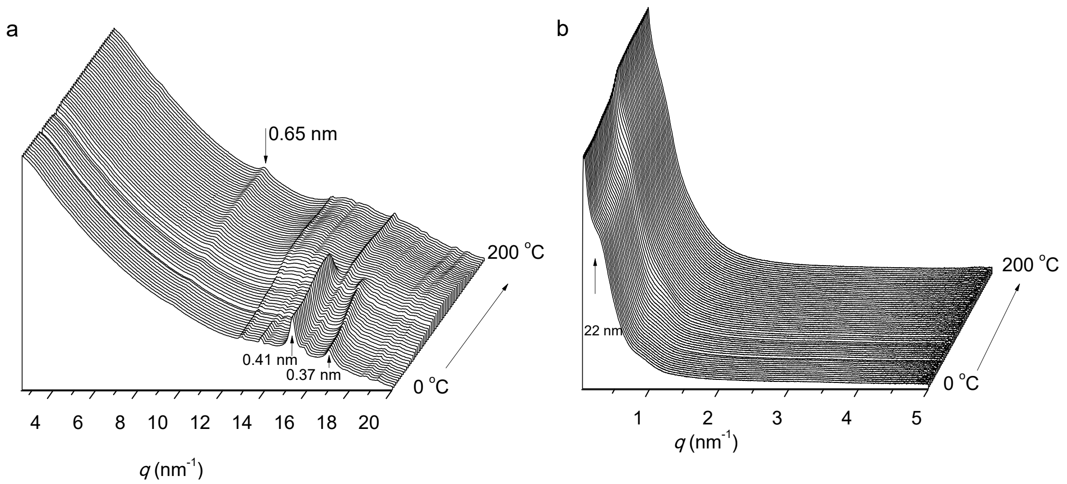

Figure S11. Evolution of the WAXS (a) and SAXS (b) profiles recorded from P[(Gl$_{48}$-*r*-PDL$_{52}$)-*g*-(LGA)$_2$] copolymer at heating over the 0-200 ºC range.

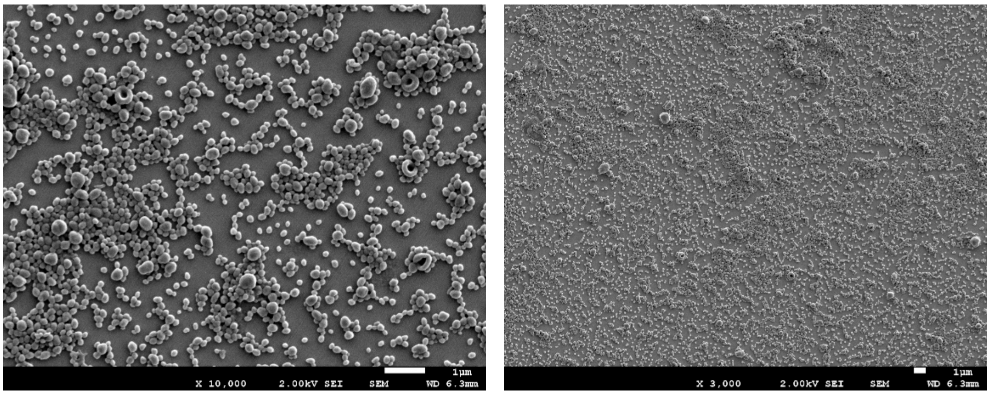

Figure S12. SEM images of nanoparticles made of P[(Gl13-*r*-PDL87)-*g*-(BLG)10].

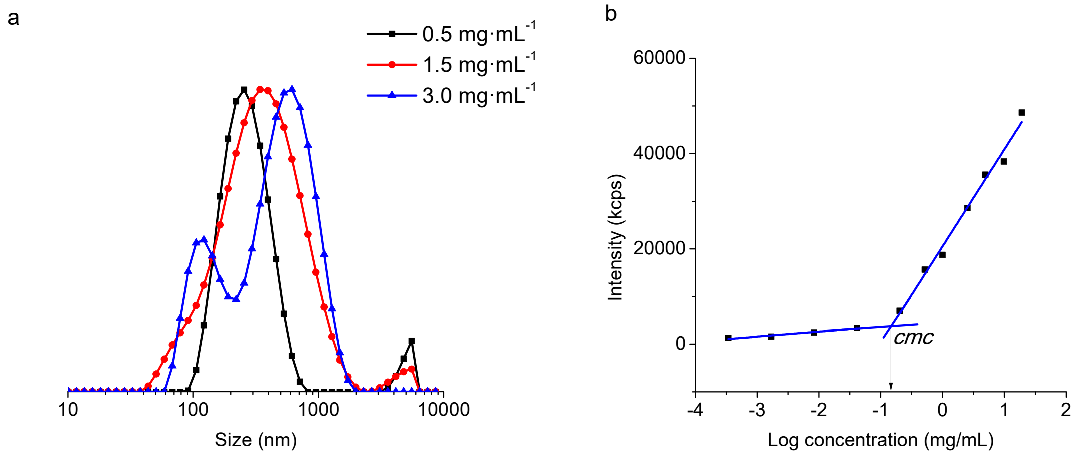

Figure S13. DLS profiles (a) and plot used for determining the critical concentration (b) of micelles made of P[(Gl13-*r*-PDL87)-*g*-(LGA)10].

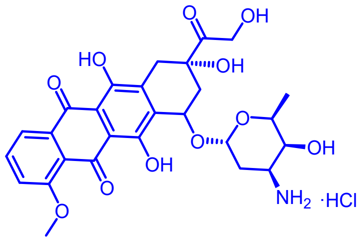

Figure S14. Chemical structure of DOX·HCl.
